# Molecular Dynamics Simulation and the Regeneration and Diffusion Effects of Waste Engine Oil in Aged Asphalt Binder

**DOI:** 10.3390/ma17102212

**Published:** 2024-05-08

**Authors:** Yuxuan Sun, Augusto Cannone Falchetto, Fan Zhang, Di Wang, Wei Chen

**Affiliations:** 1 Department of Civil Engineering, Aalto University, 02150 Espoo, Finland; yuxuan.sun@aalto.fi (Y.S.); fan.3.zhang@aalto.fi (F.Z.); 2 Department of Civil Engineering, University of Ottawa, Ottawa, ON K1N 6N5, Canada; dwang6@uottawa.ca; 3 College of Transportation Engineering, Tongji University, Shanghai 201804, China; chenwei13572468@163.com

**Keywords:** molecular dynamics, waste engine oil (WEO), regeneration mechanism, mutual diffusion

## Abstract

In recent years, the potential of waste engine oil (WEO) as a rejuvenator for aged asphalt binders has gained significant attention. Despite this interest, understanding WEO’s regeneration mechanism within aged asphalt binders, particularly its diffusion behavior when mixed with both aged and virgin asphalt binders, remains limited. This study adopts a molecular dynamics approach to constructing models of virgin, aged, and rejuvenated asphalt binders with different WEO contents (3%, 6%, 9%, and 12%). Key properties such as the density, glass transition temperature, cohesive energy density, solubility parameter, viscosity, surface free energy, fractional free volume, and diffusion coefficient are simulated. Additionally, models of rejuvenated asphalt binder are combined with those of aged asphalt binder to investigate mutual diffusion, focusing on the impact of WEO on the relative concentration and binding energy. The findings reveal that WEO notably decreased the density, viscosity, and glass transition temperature of aged asphalt binders. It also improved the molecular binding within the asphalt binder, enhancing crack resistance. Specifically, a 9% WEO content can restore the diffusion coefficient to 93.17% of that found in virgin asphalt binder. Increasing the WEO content facilitates diffusion toward virgin asphalt binders, strengthens molecular attraction, and promotes the blending of virgin and aged asphalt binders.

## 1. Introduction

Engine oil, renowned for its superb lubricating properties, is widely used in internal combustion engines [1,2,3]. However, during engine operation, engine oil becomes contaminated by combustion residues, leading to a gradual deterioration in performance. Therefore, it is essential to change the engine oil regularly for internal combustion engines. Recent data show that there are approximately 1.47 billion vehicles worldwide, with each engine requiring an oil change every 5000–7500 km [4,5]. It is estimated that in 2024, this will lead to the accumulation of 22.70 billion liters of waste engine oil (WEO) [6]. This used oil then needs to be thoroughly drained and properly disposed. In addressing the disposal of WEO responsibly, current recycling practices such as pyrolysis technology for fuel production [7,8] and refinement for lubricant reuse [9,10] offer sustainable pathways. Additionally, WEO has been proven to serve as a binder in the production of roofing tiles [11].

With the widespread use of reclaimed asphalt pavement (RAP) in engineering projects, there is a growing demand for asphalt recycling agents (ARAs) [12,13,14]. WEO, with its primary constituents of aromatic solvents, paraffin oil, and polyolefin oil, possesses the ability to effectively replenish the lightweight fractions (aromatic and saturate) that are reduced due to the aging of asphalt binder [15]. Given its potential, WEO has increasingly captured the interest of researchers. Experimental studies [16,17] have shown that WEO can effectively increase penetration, reduce the softening point, and decrease the viscosity of aged asphalt binders, gradually restoring the physical properties of these binders to the levels of virgin asphalt binders. Qurashi and Swamy [18] discovered that incorporating WEO at a proportion of 2–4% by weight of the aged asphalt binder resulted in the most significant viscosity reduction. When the addition of WEO ranged from 7 to 13%, the rejuvenation effect was equivalent to that of commercial ARAs [19]. Chen et al. [20] reported that using WEO to rejuvenate RAP in asphalt mixtures improved performance both at high and low temperatures. Farooq et al. [21] indicated that the inclusion rate of RAP could increase from a maximum of 20% to 60% with the application of WEO. However, WEO can also have some adverse effects on asphalt mixtures, such as reducing the adhesion between the asphalt and aggregate and increasing the moisture susceptibility of the mixture [22]. Moreover, studies have indicated that when the WEO content exceeds 10%, both the high-temperature stability and the fatigue performance of the asphalt mixture decrease [23]. When the WEO content surpasses 15%, the asphalt mixture also faces issues involving low-temperature cracking [24].

Although numerous macroscopic experimental methods have been developed to evaluate the rejuvenating properties of WEO, understanding how WEO improves the performance of aged asphalt binders at the molecular level, as well as its impact on diffusion between the virgin and aged asphalt binders, continues to pose challenges for researchers. The diffusion behavior within asphalt binders has been characterized at the microscopic level using techniques such as 3D fluorescence image technology (3D-FIT) [25,26], Fluorescence microscopy (FM) [27,28,29], and atomic force microscopy (AFM) [30,31]. However, the reliability of these methods largely depends on the level of sample preparation and whether the samples are mixed uniformly. Additionally, these approaches fail to explain the rejuvenation mechanisms of aged asphalt binder adequately.

Molecular dynamics simulation (MD) software “Materials Studio 2020” can accurately simulate interactions between the atoms and molecules within materials and has been extensively applied in asphalt binder simulations [32,33,34,35]. Previous research has validated its precision in modeling the aging and rejuvenation processes of asphalt binders [36,37,38]. Yan et al. [39] conducted a systematic study on the rejuvenating behavior of aged asphalt binder with 14 common rejuvenators. The results found that rejuvenators with weaker polarity are more conducive to rejuvenating aged asphalt molecules, which could affect the diffusion behavior between molecules. Meng et al. [40] developed a molecular dynamics model for the diffusion system of rejuvenated asphalt. They determined that molecular forces and micro-voids promote the diffusion of asphalt molecules and rejuvenators. Zhan et al. [41] discovered that raising the temperature effectively promotes the fusion between virgin and aged asphalt molecules. Ding et al. [42] noted that adding rejuvenator molecules to the aged asphalt binder model significantly affected its diffusion coefficient more than adding them to the virgin asphalt binder model. Sun et al. [43] analyzed rejuvenators with four distinct molecular structures and concluded that the cyclic aliphatic-aromatic compounds demonstrated superior diffusion performance in aged asphalt binders.

Although there has been research on the rejuvenating effects of aged asphalt binders and the diffusion processes between virgin and aged binders, most studies have concentrated on rejuvenators composed of simple aromatic or saturated molecules. There is a relative lack of research on complex rejuvenators composed of multiple molecules, such as WEO. Furthermore, there is a need for more in-depth explanations at the molecular level on how the dosage of WEO affects the rejuvenation behavior. Therefore, it is essential to conduct research addressing these gaps, which hold significant importance in the field of asphalt recycling and aged asphalt binder rejuvenation, with particular focus on enhancing rejuvenation techniques, optimizing rejuvenator usage, and improving the molecular fusion between virgin and aged asphalt binders.

## 2. Objective and Scope

The present study aims to investigate the rejuvenation mechanism of waste engine oil in aged asphalt binders, specifically focusing on its diffusion behavior when blended with both aged and virgin asphalt binders. Utilizing molecular dynamics methods, the research establishes models for the virgin asphalt binder, aged asphalt binder, and rejuvenated asphalt binder models incorporating varying dosages of WEO. Through calculations of the density, glass transition temperature (*T_g_*), cohesive energy density (CED), solubility parameter (*δ*), viscosity (*η*), surface free energy (*γ_a_*), fractional free volume (FFV), and diffusion coefficient (*D*), this study explores the impact of the WEO dosage on the rejuvenation process and elucidates the fundamental rejuvenation mechanisms. Additionally, mutual diffusion models for virgin and rejuvenated asphalt binders with different WEO dosages are developed. This study then analyzes WEO’s effects on the relative concentration and binding energy within the mutual systems.

## 3. Molecular Models and Simulation Details

### 3.1. Construction of Simulation Models

#### 3.1.1. Asphalt Binder Models

**Figure 1:**
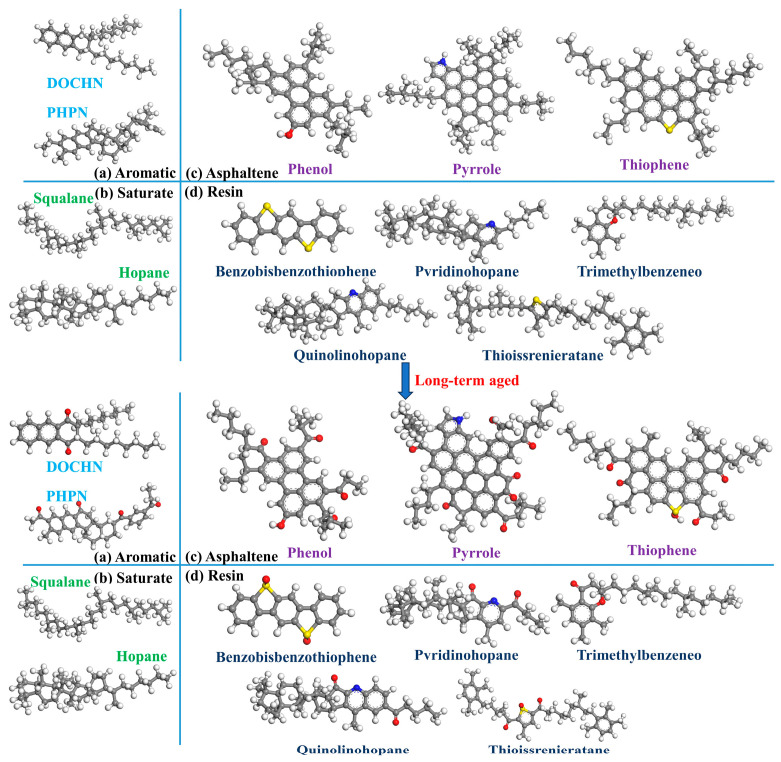
Molecular models of virgin and long-term aged asphalt binders, with color coding for the atoms as follows: carbon in gray, sulfur in yellow, hydrogen in white, nitrogen in blue and oxygen in red.

Asphalt binder, characterized by a complex and varied array of hydrocarbons, presents challenges in precisely determining its chemical structure due to its intricate composition. In this study, the AAA-1 asphalt binder model [44], including saturates, aromatics, resins, and asphaltenes (SARAs), was utilized and assembled using the amorphous module in Materials Studio (MS) 2020 software [45]. In previous studies, Fourier transform infrared spectroscopy (FTIR) analysis revealed that carbonyl and sulfoxide groups emerge as characteristic functional groups in asphalt binder following the aging process [46,47,48]. Therefore, the model for long-term aged asphalt was represented by a fully oxidized version of the 12 component model [49]. The detailed molecular models are shown in Figure 1.

Based on Qu et al.’s [50] study, the NY1 and NY3 molecular models were selected to represent the virgin and long-term aged asphalt binders, respectively. Table 1 shows the components and number of molecules in each binder.

#### 3.1.2. WEO Models

**Figure 2:**
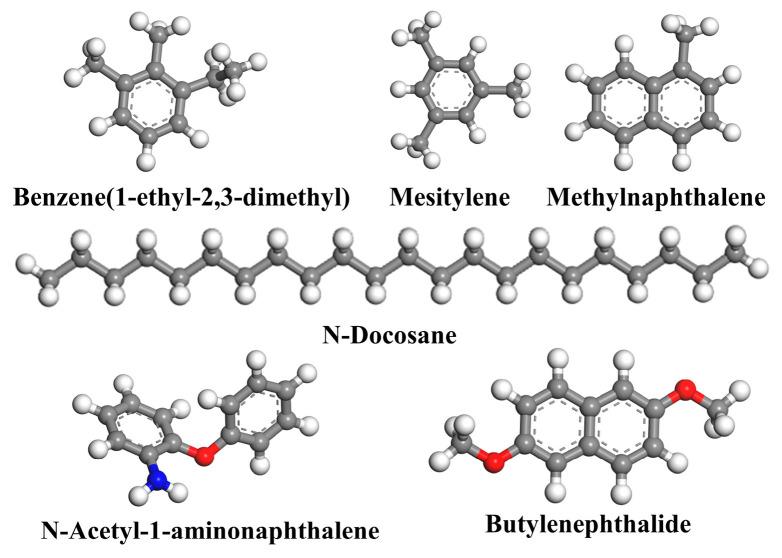
Molecular model of WEO, with color coding for the atoms as follows: carbon in gray, hydrogen in white, nitrogen in blue and oxygen in red.

By conducting a gas chromatograph mass spectrometry (GC–MS) test and gel permeation chromatography (GPC) test, the detailed components of WEO proposed by Liu et al. [15], excluding any metal impurities, are listed in Table 2. The WEO molecular model is shown in Figure 2.

#### 3.1.3. WEO-Rejuvenated Models

This study selected four different WEO dosages—3%, 6%, 9%, and 12% by weight of WEO to the aged asphalt binder—to study the rejuvenating effects of WEO. After mixing with aged asphalt binders, they are denoted as 3WEO, 6WEO, 9WEO, and 12WEO, respectively. Some studies indicate that higher proportions of WEO can potentially weaken the bonding strength between the asphalt binder and aggregates, leading to adverse effects on the performance of asphalt mixtures [16,51,52]. Therefore, in this study, the maximum WEO dosage selected was 12% to ensure a professional and balanced approach to the analysis.

#### 3.1.4. Mutual Diffusion Models

**Figure 3:**
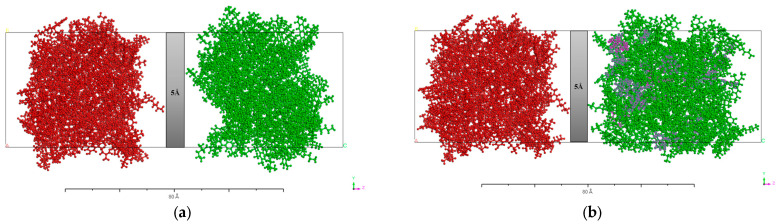
Mutual diffusion models. (**a**) Virgin—aged diffusion model (without WEO). (**b**) Virgin—12WEO diffusion model (WEO dosage of 12%). (Red represents virgin asphalt binder model, green represents aged asphalt binder model, and pink represents WEO molecules).

Rejuvenators are typically blended with the aged asphalt binder and combined with virgin asphalt to prepare the recycled mixture [53]. The diffusion behavior was investigated by assembling the virgin—virgin diffusion model, virgin—aged diffusion model (without WEO), virgin—3WEO diffusion model (WEO dosage of 3%), virgin—6WEO diffusion model (WEO dosage of 6%), virgin—9WEO diffusion model (WEO dosage of 9%), and virgin—12WEO diffusion model (WEO dosage of 12%). The virgin asphalt binder layer comprised 86 molecules in the mutual diffusion models. In comparison, the aged asphalt binder layer consisted of 79 aged asphalt molecules along with varying numbers of WEO molecules: 6, 15, 23, and 30. A 5 Å vacuum layer was introduced between the materials in the simulation to represent the physical separation that exists before molecular diffusion begins. This vacuum layer eliminates the interaction between surface atoms, accurately reflecting real-world conditions where materials are initially separated, thus providing a more realistic simulation of how substances diffuse when they come into contact [54]. Additionally, as shown in Figure 3, periodic boundary conditions without a vacuum layer were established to account for the complexities encountered in actual construction environments [55].

### 3.2. Simulation Details

**Figure 4:**
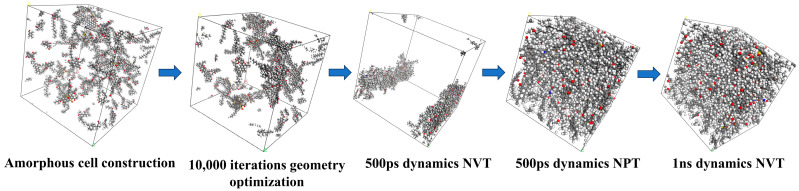
The simulation process of the aged asphalt binder molecular model.

Material Studio (MS) 2020 software was selected for modeling the molecular dynamics (MD). Firstly, molecules of asphalt and WEO were evenly distributed in a cubic box at a preliminary density of 0.1 g/cm^3^ to mitigate any overlapping or entanglement effects. This process was followed by geometry optimization, utilizing the smart descent algorithm across 10,000 iterations to achieve a stable molecular configuration. Subsequently, a dynamic simulation phase lasting 500 ps was conducted under a constant particle number, volume, and temperature (NVT) ensemble to gradually bring the molecular system to the desired temperature. This phase was followed by another 500 ps MD simulation under a constant particle number, pressure, and temperature (NPT) ensemble to facilitate the thorough mixing of the system. After 500 ps of computation, the temperature, energy, and density of the asphalt molecular system were essentially constant, thus demonstrating that they reached equilibrium under both the NVT and NPT ensembles. Finally, another 1 ns NVT MD simulation was executed, allowing for detailed calculation and evaluation of the thermal behavior of the molecules. Figure 4 illustrates the process from molecular assembly to molecular dynamics simulation, using the aged asphalt binder model as a representative example.

The COMPASS II forcefield was utilized in dynamic simulations to characterize molecular interactions. Leveraging two comprehensive molecular databases, it can search and create model molecules apt for parameterization, thereby significantly improving the simulation of heterocyclic systems, including asphalt binders [56,57]. Temperature control was achieved through the Nose–Hoover–Langevin (NHL) thermostat, while pressure was maintained at 1.0 atm using the Andersen barostat. Electrostatic interactions were addressed using the Ewald summation technique, and the atom-based summation method was adopted for the van der Waals (vdW) interactions. Meanwhile, the cut-off distance was set to 15.5 Å, which was determined by balancing the minimum image convention, simulation cost, and insights from previous studies [58]. All calculations were conducted at 433.15 K with a fixed time step of 1.0 fs to expedite the equilibrium of the asphalt molecules and enhance the blending efficiency of WEO in the asphalt binder [59,60].

A comparable sequence of dynamic simulations was performed for the mutual diffusion system, encompassing 10,000 iterations of geometry optimization, followed by 500 ps NVT, then 500 ps NPT, and concluding with a 1 ns NVT simulation.

## 4. Results and Discussion

### 4.1. Rejuvenation Effect on WEO

#### 4.1.1. Molecular Density

**Figure 5:**
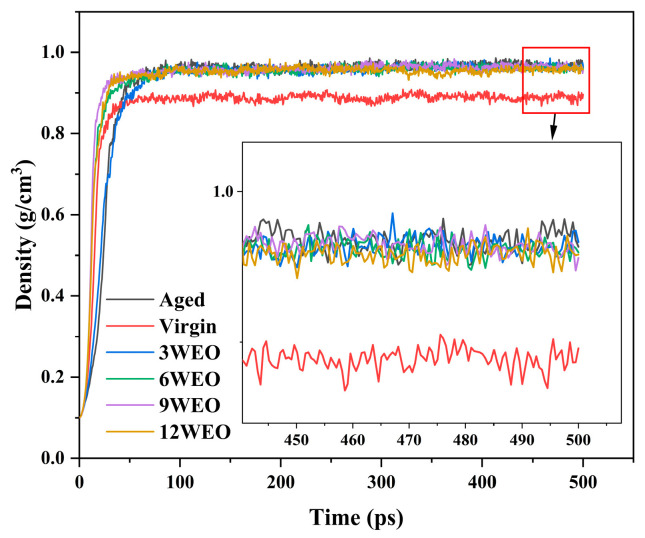
Density values of different models.

Molecular density is often considered a crucial metric for assessing the accuracy of simulation results [61]. Figure 5 illustrates the changes in density for various models under NPT simulations at 433.15 K. All of the models’ densities were observed to stabilize after the initial 75 ps. Hence, the average density calculated from 400 to 500 ps was deemed to accurately represent the asphalt molecule’s definitive density. This approach determined that the density of the virgin asphalt binder model was 0.889 g/cm^3^, while the aged asphalt binder model showed a density of 0.997 g/cm^3^. These values are considerably below those reported in the literature [62], which may be attributed to the elevated simulation temperature of 433.15 K. High temperatures can lead to thermal expansion and a resultant decrease in density, explaining why the modeled densities were lower than what is typically expected. The incorporation of WEO resulted in a decrease in the density of the aged asphalt binders. This reduction was particularly noticeable with the addition of 12% WEO, where the density reached its lowest at 0.958 g/cm^3^. WEO could compensate for the lack of lighter components in aged asphalt binders, thereby leading to a reduction in density.

#### 4.1.2. Glass Transition Temperature

**Figure 6:**
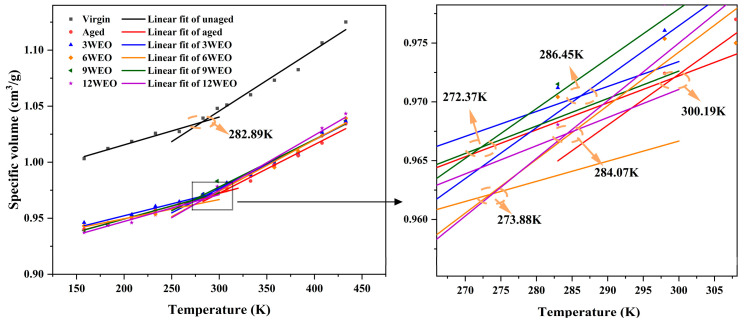
*T_g_* values of different models.

The glass transition temperature (*T_g_*) plays a crucial role in defining the physical characteristics of asphalt materials, reflecting their performance under low-temperature conditions. In this study, the inverse of the densities of the asphalt models within different temperature ranges was linearly fitted. *T_g_* was determined by identifying the temperature corresponding to the intersection point of adjacent fitted lines. NPT simulations of 300 ps were conducted on the virgin, aged, and WEO-regenerated asphalt models to assess the effect of WEO on restoring the glass transition behavior in aged asphalt. These simulations spanned a temperature range from 158.15 K to 433.15 K, with intervals of 25 K. The results, illustrated in Figure 6, were calculated based on the last 50 ps of the simulations.

The findings presented in Figure 6 reveal that the *T_g_* of the aged asphalt binder model exceeded that of the virgin asphalt binder model, rising from 282.89 K to 300.19 K. This increase stemmed from changes in the asphalt’s compositional balance during aging and the conversion of some molecules into larger molecular weights. Consequently, this enhanced the intermolecular forces in the asphalt, thereby restricting molecular mobility. More energy was thus required to shift from the glassy state to the high-elastic state, resulting in an elevated temperature threshold for this transition. However, incorporating WEO into the asphalt increased the molecular chains’ flexibility, which in turn weakened the intermolecular forces. As such, the *T_g_* of all WEO-regenerant asphalt binder models was observed to be lower than that of the aged asphalt. Among these, 9WEO demonstrated the most advantageous low-temperature performance with a *T_g_* of 272.37 K. This was followed closely by 6WEO at 273.88 K, 12WEO at 284.07 K, and 3WEO at 286.45 K.

#### 4.1.3. Cohesive Energy Density, Solubility Parameter, and Viscosity

The cohesive energy density (*CED*) acts as a vital parameter for assessing the intermolecular binding robustness in an asphalt molecular framework. It quantifies the amount of energy needed for each unit of volume to disperse the molecules, thus indicating the intrinsic cohesive force and molecular interplay within the asphalt binders [61]. Meanwhile, it is often used alongside the solubility parameter (*δ*), which emerges as a tool for examining the solvent–solute dynamics within asphalt binders. The value of *δ* comprises two components: the van der Waals force (*δ_vdw_*) and electrostatic interaction (*δ_ele_*). Additionally, the value of *δ* can also be calculated by taking the square root of the *CED* value. The detailed calculation methods for the *CED* and *δ* are shown in Equations (1) and (2), respectively:

(1) $$CED=\frac{{-E}_{inter}}{V}=\frac{E_{intra}-E_{total}}{V}$$

(2) $$\delta =\sqrt{CED}=\sqrt{{\left(\delta_{vdw}\right)}^{2}+{\left(\delta_{ele}\right)}^{2}}$$

where *E_inter_* represents the cumulative intermolecular energy; *E_intra_* represents the cumulative intramolecular energy; and *E_total_* represents the total system energy.

Viscosity is an indicator of the asphalt binder model’s resistance to shear forces. On the nanoscale, it can reflect its molecular structure, while on the macroscale, it manifests as the magnitude of its fluidity. Using the shear function in the Focite module, the viscosities of different asphalt models were calculated at a shear rate of 1 ps^−1^ and a temperature of 433.15 K, with the simulation time set to 1 ps.

Table 3 presents the trends of the *CED*, *δ*, and *η* values with the aging of asphalt and the addition of WEO. Upon aging, the increase in ketones and sulfoxides enhanced the polarity among molecules, significantly boosting the intermolecular forces per unit volume and diminishing fluidity. Consequently, this resulted in higher *CED*, *δ*, and *η* values. However, the values of the *CED*, *δ*, and *η* decreased with the addition of WEO, indicating that WEO could integrate well with the aged asphalt binder model. The light components in WEO effectively weakened the intermolecular forces of the aged asphalt molecules, acting as a lubricant. Among these, the effect on *η* was the most significant. When 12% WEO was added to the aged asphalt binder, there was a reduction of 10.77% compared with the aged asphalt binder without WEO. However, the results indicated that 12% WEO may disproportionately influence the dynamics of the asphalt binder, affecting its thermal properties differently compared with lower concentrations. This could be due to excessive WEO molecules introducing more complex molecular interactions. These interactions can create a more tangled molecular environment, which may increase the cohesion among the molecules.

#### 4.1.4. Surface Free Energy

**Figure 7:**
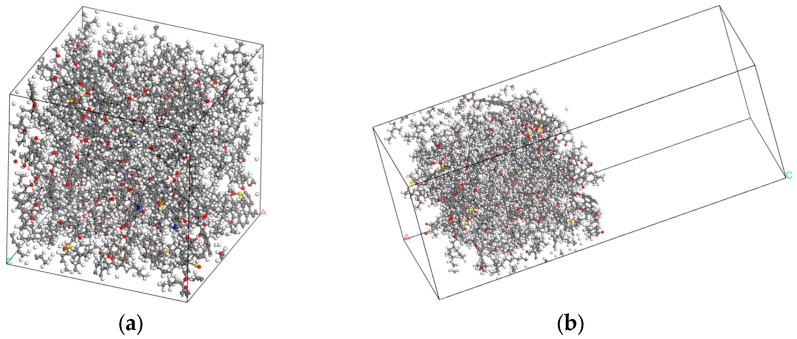
Aged asphalt binder’s *γ_a_* model. (**a**) The bulk asphalt binder model. (**b**) The confined asphalt binder model.

Surface free energy (*γ_a_*) is an indicator of the crack resistance performance of asphalt binders. It refers to the energy necessary to create a new unit surface area of material under vacuum conditions [63]. Before calculating this value, the confined and bulk asphalt binder models needed to be established first. By setting a 50 Å vacuum layer in the OZ direction, the confined asphalt binder models no longer possessed periodicity. Figure 7 illustrates the aged asphalt binder’s *γ_a_* model.

The cohesion work (*W_aa_*) refers to the energy needed to split a unit area into two separate sections, and its numerical value is twice that of *γ_a_*. For asphalt molecules, it can measure the amount of work needed for internal damage [64]. The equations for *γ_a_* and *W_aa_* are shown below:

(3) $$\gamma_{a}=\frac{E_{film}-E_{bulk}}{2A}$$

(4) $$W_{aa}=2\gamma_{a}$$

where *E_flim_* and *E_intra_* represent the potential energy of the confined and bulk asphalt models, respectively, and *A* represents the area of the new surface to be formed.

**Figure 8:**
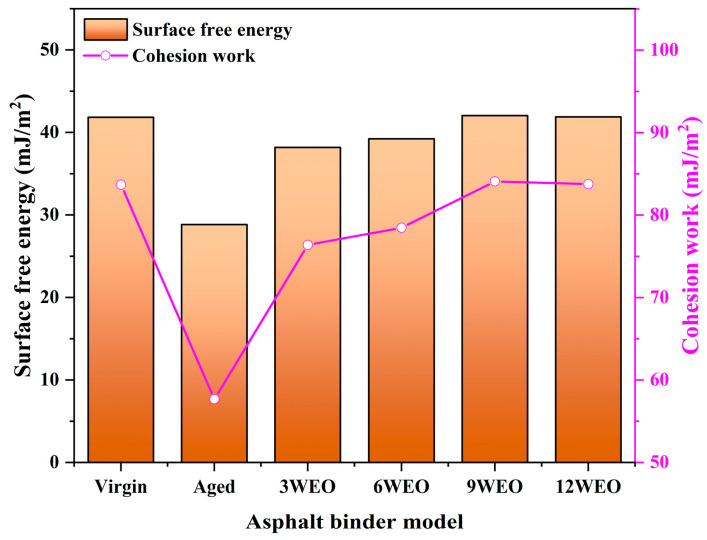
The *γ_a_* and *W_aa_* values for different models.

Figure 8 presents the *γ_a_* and *W_aa_* values for different asphalt binder models. After aging, the *γ_a_* and *W_aa_* values decreased significantly, showing a decline of 36.28% compared with the virgin asphalt binder model. This indicates that aging weakened the internal cohesion of the asphalt molecules, making them more prone to separation and, on a macroscopic level, making the asphalt binders more susceptible to cracking. Studies have shown that an increase in the asphaltene ratio is a crucial factor contributing to weakening cohesion within aged asphalt molecules. With the addition of WEO, the values of *γ_a_* and *W_aa_* gradually increased. Introducing lighter components such as resin effectively could improve the *γ_a_* and *W_aa_* values. When the WEO content reached 9%, it essentially restored to the level of virgin asphalt binder.

#### 4.1.5. Fractional Free Volume

The fractional free volume (*FFV*) is defined at the atomic level as it influences the mobility and permeability of asphalt molecules after aging and regeneration, elucidating their diffusion and glass transition behaviors [65]. The total molecular volume (*V_total_*) consists of the free volume (*V_free_*) and occupied volume (*V_occupied_*). *V_free_* allows for the free movement of molecules within it, facilitating the flow of asphalt molecules. By measuring the Connolly surface with hard spherical probes (HSPs), the size of *V_occupied_* can be determined, allowing for calculation of the *FFV* [66]. The equation is as follows:

(5) $$FFV=\frac{V_{total}-V_{occupied}}{V_{total}}=\frac{V_{free}}{V_{total}}$$

**Figure 9:**
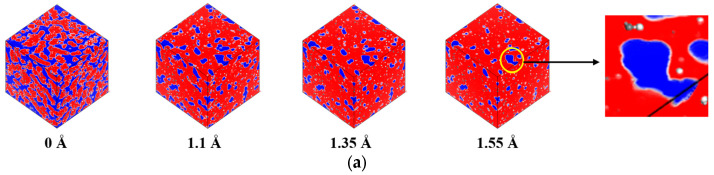

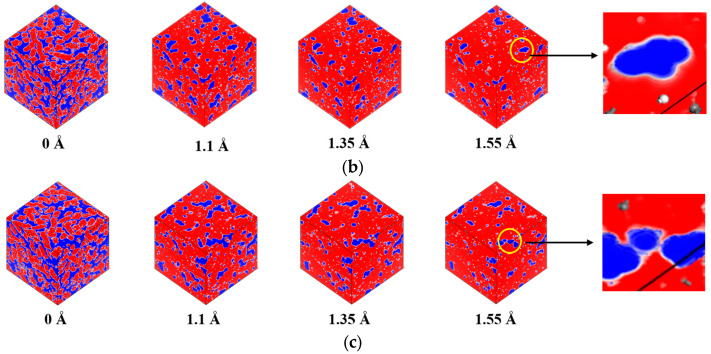
Free volume and occupied volume distributions at 433.15 K. (**a**) Virgin asphalt binder model. (**b**) Aged asphalt binder model. (**c**) Aged asphalt binder model with 12% WEO. (Blue represents free volume; red represents occupied volume; and grey represents Connolly surface).

To investigate the size effects of probe atoms, this study selected three atomic probe radii: 1.1 Å, 1.35 Å, and 1.55 Å, representing hydrogen, carbon, and oxygen, respectively. Additionally, to obtain the *V_free_* of the asphalt model under ideal conditions, a probe with a radius of 0 Å was also used. The calculations also considered room temperature to be 298.15 K and the mixing temperature to be 433.15 K to examine the impact of temperature on the *FFV*. Figure 9 displays the distribution of *V_free_* and *V_occupied_* in the virgin asphalt binder model, aged asphalt binder model, and aged asphalt binder model with 12% WEO at 433.15 K using different probe atoms.

**Figure 10:**
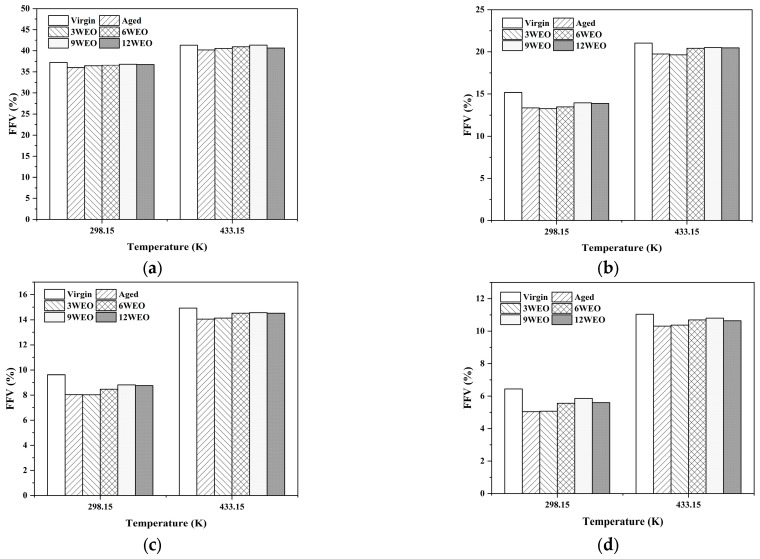
The FFV values at different WEO dosages under various probe radii and temperatures. Probe radiii are (**a**) 0 Å; (**b**) 1.1 Å; (**c**) 1.35 Å; and (**d**) 1.55 Å.

Figure 10 depicts that WEO can increase the *FFV* of aged asphalt molecules. This is attributed to the presence of chain alkanes in WEO, which can create certain void spaces within the molecular structure. Simultaneously, WEO introduces new aromatic compounds whose π-π stacking interactions can make the molecular packing denser, ultimately improving the *FFV* of aged asphalt molecules. With an increase in the probe radius, the detection range gradually decreased, leading to a downward trend in the *FFV*. However, the *FFV* increased with the rising temperature, which is consistent with the result that asphalt binder flows and diffuses more easily at higher temperatures [67].

#### 4.1.6. Diffusion Coefficient

The impact of rejuvenators on the diffusion characteristics of aged asphalt molecules can be accurately assessed by measuring the passage rate of molecules per unit area at a unit concentration gradient through mean square displacement (*MSD*) calculations. This coefficient is determined using the Einstein equation [68]. In NVT ensemble calculations, the total number of molecules *N* remains constant. Therefore, when the *MSD* curve stabilizes and shows a linear trend over a specific simulation time, its slope represents the diffusion coefficient (*D*). The detailed equation is as follows:

(6) $$D=\frac{1}{6}MSD=\frac{1}{6N}\underset{t\to \infty }{\lim}\frac{d}{dt}\sum_{n=1}^{N}<{\left[r_{i}\left(t\right)-r_{i}\left(0\right)\right]}^{2}>$$

where *N* represents the overall count of molecules present in the model and *r_i_*(0) and *r_i_*(*t*) represent the positions of molecule *n* at the initial moment and after moving at time *t*, respectively.

**Figure 11:**
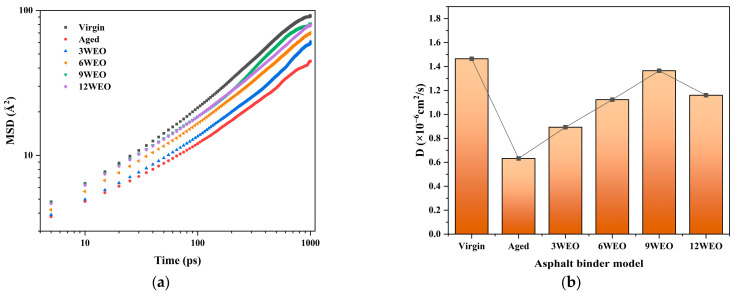
*MSD* and *D* values of different models. (**a**) *MSD* values. (**b**) *D* values.

Figure 11 illustrates the *MSD* curves of different asphalt binder models at 433.15 K. After aging, the *MSD* values decreased significantly, indicating a slower diffusion rate. However, with the addition of WEO and an increase in its dosage, the *MSD* curves gradually approached the level of the virgin asphalt binder model. Due to the effects of the periodic boundary conditions, interactions between the model boundaries and external molecules altered the *MSD* curve slopes at the end of the *MSD* curves.

**Figure 12:**
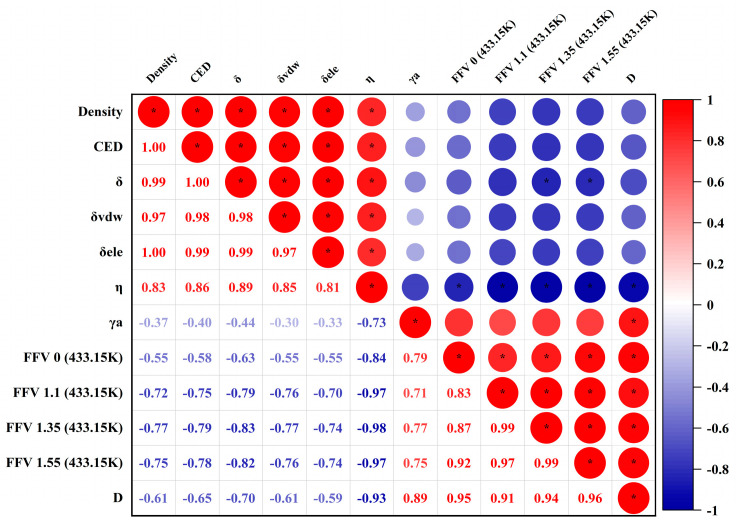
Correlation analysis of different models (“*” represents the significance level *α* ≤ 0.05).

Figure 12 calculates the *D* values for each asphalt binder model. The *D* value for the aged asphalt binder model was only 39.58% of that of the virgin asphalt binder model. The most significant recovery of *D* was observed with the addition of 9% WEO, reaching 93.17% of the virgin asphalt binder model’s value. However, achieving 100% diffusion was not possible due to limitations in molecular compatibility and simulation constraints. As the WEO content increased, the recovery rate of *D* slowed down, especially when the WEO content reached 12%, at which point its *D* value decreased. This finding aligns with previously published research in the literature [55].

#### 4.1.7. Correlation Coefficient Matrix

Figure 12 presents a heatmap of correlation coefficients detailing the interrelationships among various parameters within the computational analysis. It explores how these parameters interacted and potentially influenced each other. A pronounced positive correlation existed between the density and parameters like the *CED*, *δ*, *δ_vdw_*, and *δ_ele_*, as evidenced by the intense red coloration and coefficient values approaching 1.0. The property *η* displayed a moderate-to-strong positive correlation with the density, *CED*, *δ*, *δ_vdw_*, and *δ_ele_*, underscored by the red hues and values surpassing 0.8. In contrast, the *γ_a_* showed a pronounced negative correlation with the *FFV* across different probe radii. Notably, the *D* value correlated quite strongly and positively with the *FFV* at probe radii of 1.35 and 1.55 Å while inversely correlating with the *γ_a_*. This pattern suggests that increased FFVs at specified probe radii are indicative of elevated diffusion rates.

### 4.2. Diffusion Effect and WEO

#### 4.2.1. Relative Concentration Analysis

The relative concentration (*R*) is determined by comparing the density of atoms in each section to the overall atom density in the entire unit cell. It can simulate the degree of fusion of different asphalt binder models, thereby determining the effect of WEO on the fusion. The equation is as follows:

(7) $$R=\frac{N_{i}{/V}_{i}}{N/V}$$

where: *N_i_* represents the count of atoms in a unit *i*; *V_i_* represents the volume of unit *i*; *N* represents the overall count of atoms in the cell; and *V* represents the whole volume of the cell.

**Figure 13:**
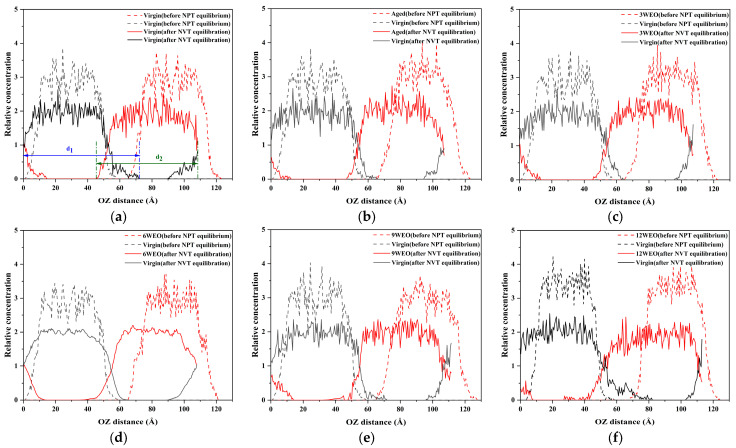
Relative concentration at 433.15 K. (**a**) Virgin—virgin diffusion model. (**b**) Virgin—aged diffusion model. (**c**) Virgin—3WEO diffusion model. (**d**) Virgin—6WEO diffusion model; (**e**) Virgin—9WEO diffusion model; (**f**) Virgin—12WEO diffusion model.

Figure 13 shows the *R* values of the virgin asphalt binder model at 433.15 K when mixed with another virgin asphalt binder model, an aged asphalt binder model, and aged asphalt binder models with varying WEO contents to form blended systems. Initially, gaps existed between the asphalt molecules, but these gaps disappeared after 500 ps of NVT equilibration. At the same time, the peak values decreased, and the area of overlap in the relative concentration increased, suggesting enhanced molecular integration. Incorporating aged asphalt binder reduced the overlap range with the virgin asphalt binder, shrinking from 45–71 Å to 47–65 Å. However, as the amount of WEO increased, the overlap range gradually expanded, with their ranges being 45–64 Å, 34–66 Å, 43–67 Å, 32–71 Å, and 28–82 Å. This suggests that WEO promotes integration between aged and virgin asphalt molecules.

**Figure 14:**
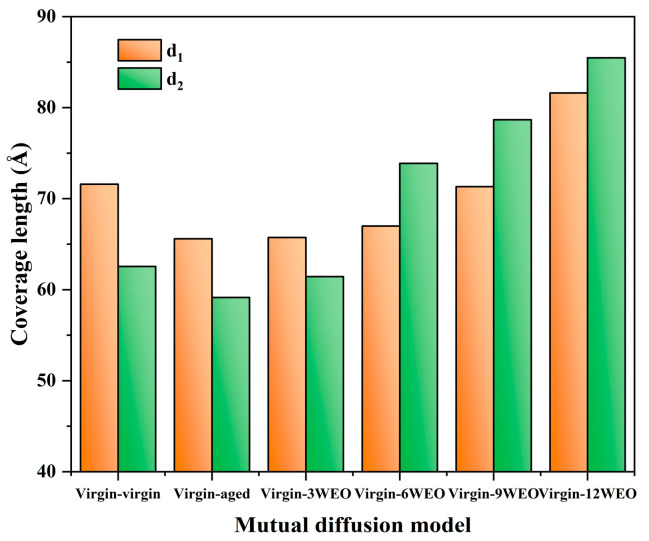
Coverage lengths.

To assess the diffusion behavior of asphalt molecules within the blended systems, *d_1,_* and *d_2_* are defined to represent the coverage lengths of the two molecular types present in the system. Figure 13a marks the respective coverage lengths of two virgin asphalt molecules. The calculated coverage lengths of different asphalt molecules under various fusion systems are shown in Figure 14. When the virgin asphalt molecule was mixed with the aged asphalt molecule, as opposed to mixing with itself, there was a decrease in the virgin asphalt molecule’s coverage length. This reduction is attributed to the compositional differences between the new and aged asphalt molecules, which diminished their ability to diffuse effectively. This phenomenon predominantly showed the virgin asphalt molecule diffusing toward the aged asphalt molecule. As the WEO content increased from 3% to 12%, the coverage length of the virgin asphalt molecules increased by 0.21%, 2.12%, 8.72%, and 24.41%, while the coverage length of the aged asphalt molecules increased by 3.89%, 24.92%, 33.04%, and 44.54%, respectively. Due to the addition of virgin asphalt binder, the concentration of the WEO molecules within the entire blended system was reduced. These findings suggest that as the WEO content increases, the effect of WEO on enhancing the diffusion of aged asphalt molecules into virgin asphalt molecules becomes more pronounced.

#### 4.2.2. Binding Energy

According to a study by Liu et al. [69], binding energy can be used to express the attractive and repulsive forces between molecules, thereby assessing their diffusion capability. When the binding energy is positive, the molecules attract each other, and when it is negative, they repel each other. Equation (8) outlines the method for calculating the binding energy:

(8) $$E_{binding}=E_{a}+E_{b}-E_{ab}$$

where *E_a_* represents the energy of molecule *a*; *E_b_* represents the energy of molecule *b*; and *E_ab_* represents the energy of the blended system *ab*.

**Figure 15:**
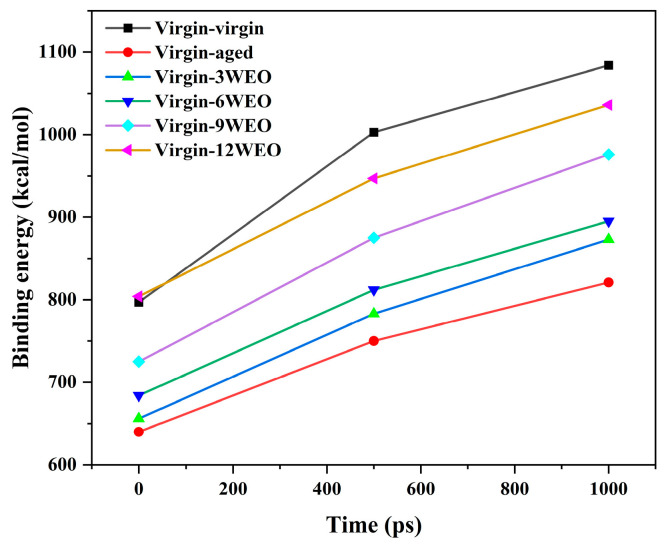
Binding energies in the mutual diffusion models.

Figure 15 presents the binding energies at the onset (0 ps), midpoint (500 ps), and conclusion (1 ns) of NVT equilibration in the mutual diffusion models. The binding energies were consistently positive throughout these stages, indicating attractive forces between the molecules. The binding energy between the virgin and aged asphalt binders showed a 28.28% increase over time. However, in contrast to the self-blended virgin asphalt binder model, the binding energy between the aged and virgin asphalt binders decreased. This decrease is attributed to the presence of aged asphalt binder, which increases electrostatic interactions between molecules, preventing full integration between the virgin and aged asphalt binders. The incorporation of WEO led to increases in the binding energy at 1 ns by 6.33%, 9.01%, 10.35%, and 26.19% for different WEO contents compared with the mixture without WEO. Significantly, introducing 12% WEO into the aged asphalt binder at 0 ps achieved a binding energy surpassing that of the virgin asphalt binder blended with itself. However, as the equilibration process unfolded, this binding energy diminished, ultimately falling below the level observed in the self-blended virgin asphalt binder model. Although WEO could improve the binding energy, it still performed worse than the self-blended virgin asphalt binder model. As the simulation time increased, the growth rate of the binding energy in each model decreased, highlighting that mechanical stirring is necessary for more thorough mixing of the virgin and aged asphalt binders.

## 5. Conclusions

This research used molecular dynamics simulation to explore the rejuvenating effects of waste engine oil (WEO) on aged asphalt binders and its impact on the diffusion behaviors between virgin and aged asphalt molecules. The analysis encompassed the binders’ fundamental physical and thermodynamic properties, fractional free volume (*FFV*), and diffusion coefficients (*D*), alongside examining the diffusion behaviors and binding energy within the fusion system. The key findings are summarized below:

(1) The incorporation of WEO into the aged asphalt binder effectively decreased its density. This rejuvenation also led to a noticeable reduction in the glass transition temperature (*T_g_*), enhancing the asphalt binder’s performance under lower temperature conditions.
(2) WEO could reduce the cohesive energy density (*CED*), solubility parameter (*δ*), and viscosity (*η*) of the aged asphalt binder. Moreover, WEO significantly restored the surface free energy (*γ_a_*) and cohesive work (*W_aa_*) of aged asphalt binder, effectively restoring its cracking resistance to match that of virgin asphalt binder.
(3) The addition of WEO enriched the light components in the aged asphalt binder, improving its diffusion coefficient (*D*) by increasing the fractional free volume (*FFV*). However, WEO could not fully return its diffusion properties to the virgin binder levels, and too much WEO diminished its effectiveness in improving the aged binder’s *D* value.
(4) The mutual diffusion models highlighted the positive impact of WEO on promoting the integration of aged and virgin asphalt molecules. An increase in the binding energy was observed with the addition of WEO, indicating enhanced compatibility between different asphalt molecules.
(5) Although the level of diffusion and bonding strength in the mutual diffusion models increased with the addition of WEO, excessive WEO content weakened the regenerative performance of aged asphalt binder. Therefore, the WEO content in practical applications should not exceed 9%.

This study underscores the potential of WEO as an effective rejuvenator for aged asphalt binders, offering a sustainable approach to asphalt pavement maintenance and recycling. By improving the cohesive properties and facilitating molecular diffusion, WEO not only restores the performance of aged asphalt binders but also contributes to the durability and sustainability of asphalt pavements. However, given the diverse range of WEO types and potential impurities, this study selected only six molecules to represent the composition of WEO. The influence of other components, particularly impurities within WEO, on the rejuvenation and fusion of aged asphalt binders is still uncertain. Therefore, future research will undertake a more comprehensive investigation using a broader spectrum of WEO molecules. Additionally, subsequent studies will assess the effects of various waste oils, such as waste cooking oil (WCO), waste vegetable oil (WVO), and tall oil, at different contents and working temperatures on the regeneration and diffusion processes in aged asphalt binders.

## Figures and Tables

**Table 1:** Molecular components of virgin and long-term aged asphalt binders.

SARA Components	Molecules in Model	Virgin Asphalt Binder Model			Long-Term Aged Asphalt Binder Model		
		Molecular Formula	Number of Molecules	Weight (%)	Molecular Formula	Number of Molecules	Weight (%)
Saturate	Hopane	C_29_H_50_	7	15.9	C_35_H_62_	7	14.9
	Squalane	C_30_H_62_	7		C_30_H_62_	6	
Aromatic	PHPN	C_35_H_44_	18	42.5	C_35_H_36_O_4_	13	33.4
	DOCHN	C_30_H_46_	21		C_30_H_42_O_2_	15	
Resin	Quinolinohopane	C_40_H_59_N	2	26.0	C_40_H_56_NO_2_	2	30.2
	Thioisorenieratane	C_40_H_60_S	2		C_40_H_56_O_3_S	2	
	Trimethylbenzeneoxane	C_29_H_50_O	15		C_29_H_48_O_2_	17	
	Benzobisbenzothiophene	C_18_H_10_S_2_	3		C_18_H_10_O_2_S_2_	4	
	Pvridinohopane	C_36_H_57_N	2		C_36_H_53_NO_2_	2	
Asphaltene	Phenol	C_42_H_54_O	3	15.6	C_42_H_45_O_5_	4	21.5
	Pyrrole	C_66_H_81_N	2		C_66_H_67_NO_7_	3	
	Thiophene	C_51_H_62_S	4		C_51_H_54_O_5_S	4	

**Table 2:** Molecular components of WEO.

Rejuvenator Type	Molecules in Model	Molecular Formula	Weight (%)
Waste engine oil	N-Docosane	C_22_H_46_	8.6%
	Mesitylene	C_9_H_12_	14.1%
	Benzene(1-ethyl-2,3-dimethyl)	C_10_H_14_	23.5%
	Methylnaphthalene	C_11_H_10_	30.2%
	N-Acetyl-1-aminonaphthalene	C_12_H_11_NO	14.1%
	Butylenephthalide	C_12_H_12_O_2_	9.4%

**Table 3:** The trends of the *CED*, *δ*, and *η* values.

Properties	Virgin	Aged	3WEO	6WEO	9WEO	12WEO
*CED* (10^8^ J/m^3^)	2.542	3.204	3.191	3.177	3.146	3.153
*δ* ((J/cm^3^)^1/2^)	15.945	17.690	17.673	17.563	17.420	17.431
*δ_vdw_* ((J/cm^3^)^1/2^)	15.685	16.433	16.628	16.535	16.418	16.421
*δ_ele_* ((J/cm^3^)^1/2^)	1.048	5.932	5.988	5.921	5.823	5.832
*η* (cP)	0.839	1.031	1.017	0.965	0.922	0.920

## Data Availability

Data are contained within the article.
